# Why do health workers in rural Tanzania prefer public sector employment?

**DOI:** 10.1186/1472-6963-12-92

**Published:** 2012-04-05

**Authors:** Nils Gunnar Songstad, Karen Marie Moland, Deodatus Amadeus Massay, Astrid Blystad

**Affiliations:** 1 Centre for International Health, University of Bergen, P.O. Box 7804, 5020 Bergen, Norway; 2 Faculty of Health and Social Sciences, Bergen University College, P.O. Box 7030, 5020 Bergen, Norway; 3 Community Social and Economic Empowerment (COSEE), P.O. Box 137, Mbulu Manyara, Tanzania; 4 Department of Public Health and Primary Health Care, University of Bergen, P.O. Box 7804, 5020 Bergen, Norway

**Keywords:** Pension benefits, Working conditions, Human resources, Rural health services, Tanzania

## Abstract

**Background:**

Severe shortages of qualified health workers and geographical imbalances in the workforce in many low-income countries require the national health sector management to closely monitor and address issues related to the distribution of health workers across various types of health facilities. This article discusses health workers' preferences for workplace and their perceptions and experiences of the differences in working conditions in the public health sector versus the church-run health facilities in Tanzania. The broader aim is to generate knowledge that can add to debates on health sector management in low-income contexts.

**Methods:**

The study has a qualitative study design to elicit in-depth information on health workers' preferences for workplace. The data comprise ten focus group discussions (FGDs) and 29 in-depth interviews (IDIs) with auxiliary staff, nursing staff, clinicians and administrators in the public health sector and in a large church-run hospital in a rural district in Tanzania. The study has an ethnographic backdrop based on earlier long-term fieldwork in Tanzania.

**Results:**

The study found a clear preference for public sector employment. This was associated with health worker rights and access to various benefits offered to health workers in government service, particularly the favourable pension schemes providing economic security in old age. Health workers acknowledged that church-run hospitals generally were better equipped and provided better quality patient care, but these concerns tended to be outweighed by the financial assets of public sector employment. In addition to the sector specific differences, family concerns emerged as important in decisions on workplace.

**Conclusions:**

The preference for public sector employment among health workers shown in this study seems to be associated primarily with the favourable pension scheme. The overall shortage of health workers and the distribution between health facilities is a challenge in a resource constrained health system where church-run health facilities are vital in the provision of health care in rural areas and where patients tend to prefer these services. In order to ensure equity in distribution of qualified health workers in Tanzania, a national regulation and legislation of the pension schemes is required.

## Background

The severe shortage of health workers is a well-known problem in many low-income countries. Considerable geographical imbalances in health worker distribution and intra-country 'migration' from rural to urban areas are pronounced problems [1]:xviii,[2]. WHO states that "[s]killed and motivated health workers in sufficient numbers at the right place and at the right time are critical to deliver effective health services and improve health outcomes." [3]:3. Munga and Mæstad point out the huge difference in health worker distribution between urban and rural areas in Tanzania [4]. Munga argues rural and remote districts are very disadvantaged in terms of number of health workers per capita and shortage of qualified staff [5]:41. In addition to the rural-urban movement in Tanzania, there is growing interest in the movement of health workers between the public and the church-run health sector. In Tanzania, church-run health services have been particularly important for health service delivery and run almost one-third of the health services [6]:280. Gilson et al. found that in Tanzania 90% of church-run hospitals are located in rural areas [7]:15. People tend to prefer church-run health facilities because they perceive the quality of services to be better [8,9], and church-run hospitals often attract patients from a wider area than their defined catchment area.

The quality of the health services is not determined by the number of health workers and their formal qualifications alone. Health workers' motivation to perform their work well is a factor attracting growing interest. Gilson and Erasmus argue that issues with a negative effect on motivation may also have a negative effect on retention [10]:2. A commonly-used definition of motivation is "an individual's degree of willingness to exert and maintain an effort towards organizational goals" [11]:1255. WHO defines health worker motivation as "the level of effort and desire to perform well" [1]:71. Studies of motivation of health workers have placed substantial emphasis on remuneration and other financial aspects of working conditions [12], but have also pointed out aspects such as career development, educational opportunities, hospital infrastructure, resource availability, hospital management and recognition as important in ensuring motivated staff [13]. Gilson et al. argue that health worker motivation "reflects a range of personal, organisational, and societal factors, including relationships with others" [14].

In the first decades following independence in 1961, the government of Tanzania focused on providing social services to the population [15]:141. Following the difficult economic situation in the 1970s and mid-1980s, structural adjustment programmes caused reduced funding of the public sector and this had a severe impact on the public health services [16]:62, [17]. Gilson and Erasmus point out that in the 1990s there was a 26% reduction in public sector employment [10]:52. A hiring freeze in the public sector from 1993 to 1999 [18]:14,[19]:2 had profound negative consequences on the availability of employment. It is reported that between 1995 and 2005 only 16% of health staff graduating from training institutions were employed in public service [18]:14. To many newly-educated health workers the church-run health facilities were the best option for employment during this period. In recent years, government employment has again become available, and this has opened up new opportunities regarding choice of workplace. Through the Ministry of Health and Social Welfare, the government has over the last five years actively tried to improve the staffing situation in the public health sector, and states that it is "necessary to put in place an aggressive effort ..., and to increase the pace of absorption of trained workforce to meet the outstanding gaps and attrition losses." [18]:14. The competition for qualified health workers makes it easy for health workers to change workplace and Pamba and Kahabi state that the improved working conditions in the public health facilities from 2005 onwards led to a loss of personnel in the church-run health facilities [20]:62. The Joint External Evaluation of the Health Sector in Tanzania 1999-2006 states that "[i]n the 1990s many public health workers moved to FBO [church-run] and private facilities attracted by higher salaries, better benefit packages, working environments and training opportunities. However, in recent years a reverse movement has occurred, largely due to the same factors, with the GoT now offering better employment conditions." [21]:80-81.

The government is by far the largest employer of health workers in Tanzania. The government runs 64.2% of the health facilities [22]:9 and employs 74% of the health workers [22]:2. The total number of hospitals in Tanzania is 223, comprising 89 government, 90 faith-based, 8 parastatal and 36 private hospitals [18]:28. However, the government proportionally runs a much larger share of the health centres and dispensaries than of the hospitals. The number of faith-based hospitals corresponds to the number of hospitals organised under the Christian Social Services Commission (CSSC) [23]. In the vernacular of the health workers in Tanzania, these health facilities are referred to as 'mission' or 'church' as many were established by Christian missionaries. In this article we refer to these health facilities as church-run.

In Tanzania, several social security funds have been created under different legislations and supervised by different government ministries. These funds pay the pension upon retirement and provide support in cases of disability, maternity or the death of a member of the social security fund. Health workers employed by the district councils are enrolled in the Local Authorities Pension Fund (LAPF) and health workers employed in church-run health facilities are enrolled in the National Social Security Fund (NSSF) [24]:71. The pension takes the form of a combination of a lump sum paid upon retirement and a monthly pension payment thereafter. The amount paid as pension, both the lump sum and the monthly instalments, is based on the salary level and the number of years of service.

The national policy documents of Tanzania emphasise that the shortage of health workers is a great concern. The Health Sector Strategic Plan III for the period 2009-2015 states that only 35% of the health worker positions are filled by qualified health workers [25]:11. The Primary Health Services Development Programme 2007-2017 [26]:10 and the Human Resource for Health Strategic Plan 2008-2013 [19]:11 likewise point to the severe shortage of qualified health workers. In the church-run health sector, human resource shortage is even more pronounced. Pamba and Kahabi reports that the private and church-run facilities combined, face a health professional shortage of more than 85% [20]:66. Albeit these figures do not distinguish between private and church-run facilities, the health worker shortage in the latter category is considerable.

There is scant knowledge of the considerations behind health workers' preferences for their type of workplace and their decisions related to changing workplace. Knowledge about such dynamics is important in order to design policies aimed at an equitable distribution of health workers, and to improve the utilisation of the limited resources. This article draws on qualitative data collected in a rural district in Tanzania and addresses health workers' preferences and their perceptions and experiences of the differences in working conditions between the public sector health facilities versus the church-run health facilities and how these differences are evaluated in their choice of workplace.

## Methods

### Study setting

The research on which this article is based was carried out in a rural district where the public health services consist of the district hospital, two health centres and more than 20 dispensaries. The district also has a large church-run hospital. The district serves as a case illustrating aspects of health workers' perceptions of public and church-run health facilities. With the strong presence of both church-run and public health facilities, health workers and users of the health services have substantial experience of both health sectors.

### Data collection and analysis

The formative phase of the research took place in 2007 and research topics to be pursued were identified. Documents collected during the course of the research period were systematically reviewed and national policy documents proved to be very useful in this respect. The district's Council Comprehensive Health Plan as well as annual reports from the church-run hospital also provided very useful information on local human resource constraints. After the formative phase, the data collection took place during four periods of fieldwork in 2008, 2009 and 2010. Qualitative in-depth interviews (IDIs) and focus group discussions (FGDs) were employed in the data collection. Interview- and topic guides were applied with flexibility to allow for time to be spent on emerging issues. During the course of the research the difference between the pension schemes was found to be an issue of much concern to all the research participants. In the IDIs and FGDs in the last part of the research this issue was specifically addressed.

The IDIs and FGDs were carried out at locations providing the necessary privacy to allow interviewees to speak freely. The data comprise 21 IDIs and eight FGDs (with a total of 41 participants) carried out at public health facilities in the period 2008-2010. Eight additional IDIs and two FGDs (with a total of eight participants) were carried out at the church-run hospital in the same district in 2010. The interviews were carried out by the first and third author of this article (Table 1).

**Table 1:** Overview of IDIs and FGDs per health sector

Data collection tool	Public	Church-run
IDI	21	8

FGD	8	2

The disproportionate number of IDIs and FGDs in public versus church-run health facilities reflects the emphasis on the public sector in this study. The data collection in the church-run facility was supplementary and was done in the final stage of the research to understand the dynamics between the two sectors affecting the human resource distribution.

The FGD participants comprised various categories of nursing staff with professional training, Clinical Officers (COs) and Assistant Medical Officers (AMOs). The FGDs proved very useful as FGD participants often engaged in discussing views and perceptions among themselves which contributes to the richness of the data (Tables 2 and 3).

**Table 2:** Overview over FGD participants

Category of staff (FGDs listed in chronological order)	Location	Participants	Men	Women
Medical attendants (auxiliary staff)	District hospital	7	1	6

Medical attendants (auxiliary staff)	District hospital	5	1	4

Nursing staff	District hospital	5	0	5

Nursing staff	District hospital	6	0	6

Clinicians (Assistant Medical Officers and Clinical Officers)	District hospital	6	6	0

Nursing staff	District hospital	5	0	5

Nursing staff	District hospital	3	0	3

Nursing staff	District hospital	4	0	4

Nursing staff	Church-run hospital	4	0	4

Nursing staff	Church-run hospital	4	1	3

**Table 3:** Overview over categories of research participants

	Public		Church-run	
	**IDI**	**FGD**	**IDI**	**FGD**

Assistant Medical Officer	2	5	0	0

Clinical Officer	7	1	1	0

Medical attendant	5	0	0	0

Nursing staff	4	23	2	8

Other professional staff in administrative/leadership positions	3	0	5	0

All the IDIs and FGDs were tape recorded and transcribed and translated into English by research assistants. Audio files, transcripts of audio files and research notes were managed by NVivo software (QSR International) with the purpose of systematic data management and analysis. The analysis of the material started with a thorough initial review of the interview notes and audio files at the time of the data collection. The first author checked all transcripts and verified the translation from Swahili to English. During the process a set of codes was identified to which sections of audio files and pieces of text were compiled. The main codes include perceptions and actual experiences of differences between the public and church-run sector in terms of: salary level; access to allowances on top of the salary; health facility infrastructure and resources; workload; disciplinary actions; access to further training and pension benefits.

The authors of the article have accrued work and research experience from Tanzania since the early 1990s and the study has an ethnographic backdrop based on earlier long-term fieldwork by three of the authors. The fourth author is a Tanzanian citizen with substantial experience in qualitative research. All authors speak Swahili, the lingua franca of Tanzania.

### Research ethics

The study is part of a collaborative research venture funded by the Research Council of Norway entitled *Strengthening human resources for health: A study of health worker availability and performance in Tanzania*. The National Institute of Medical Research (NIMR) in Tanzania granted ethical clearance for the project (ref. NIMR/HQ/r.8a/Vol. IX/433). Permission was also obtained from the Tanzania Commission for Science and Technology (COSTECH) (2007-59-CC-2006-193, 2008-181-ER-2006-193 and 2009-250-ER-2006-193). A letter from the Regional Administrative Secretary was presented to the district administration in the district where the research was carried out. Permission was granted in the church-run hospital by the hospital director. All informants participating in the IDIs and FGDs received information about the research both verbally and in writing before signing a consent form, complying with the regulations of the Tanzania National Health Research Forum [27]:25-30. Thus, written informed consent was obtained from the research participants before publication of this report. Neither the district nor the health facilities are mentioned by name so as to ensure anonymity.

## Results

During the years of the hiring freeze, there was limited public sector employment and many newly graduated professionals were employed by church-run health facilities. In the current situation where the public sector offers new employment opportunities and competitive retirement benefits, we sought to understand the preferences of health workers for public sector versus church sector employment, especially in rural areas, where church-run facilities are an important health care provider. We learned that while health workers generally prefer public sector employment, for a variety of reasons, they acknowledge there is better availability of supplies, drugs and equipment in the church-run health facilities. In the sections below, we will address how our informants perceive and experience the differences in working conditions between the government- and the church-run health facilities and how this impact on their decision on where to work. Many of the health workers interviewed, both in the public and in the church-run sectors, had work experience from health facilities with a different type of ownership and were thus in a position to make comparisons based on their own experiences. Among the government-employed health workers, 18 reported having moved from private, church-run or parastatal health facilities to public health facilities, and four of the interviewed health workers in the church-run hospital had work experience from public health facilities.

### Workload and working hours

All the health workers said their workload was too high, regardless of employment sector. Public-sector health workers said they viewed the workload to be higher and less flexible in the church-run health sector. Health workers in the latter sector generally agreed to this statement. One nurse who had worked in both sectors said she preferred public sector employment:

There [church-run health facilities], there is too much work in relation to the pay one gets. The workload here is less severe, there the work is too much. (Nurse, district hospital, FGD)

One Clinical Officer, currently employed in the public sector, said:

There [church-run hospital] the workload is great. When you get in, in the morning, the gate is closed until 4 pm or so. All are normally very busy without rest. But here [district hospital], in between working hours one can get a short break for tea. But there such a thing does not exist. Once you are in for work the gate is closed. (CO, district hospital, IDI)

Staff at the district hospital agreed that there is a relatively lenient regulation of working hours and a high degree of flexibility at work. Another Clinical Officer explained:

I prefer to have breakfast before starting work. When I come for the morning shift I have to report at 7.30 am, and then I take about 20 minutes for my breakfast. Then I work up to 2 pm, ready to leave for home. (CO, district hospital, IDI)

A Clinical Officer at the church-run hospital gave a corresponding view on the differences and explained:

There [district hospital] the work is less than here. They also have more freedom and shorter working hours (CO, church-run hospital, IDI)

Health workers in the church-run hospital also pointed out that their hospital receives a large number of patients who have by-passed closer public health facilities because of expectations of higher-quality services. The health workers at the church-run hospital point out that the workload is very high.

### Human resource management and disciplinary actions

Health workers in the public sector said employees are treated with more respect, have reliable access to sick leave and a voice in their workplaces than they would have at a church-run health facility. A nurse at the district hospital explained with reference to church-run health facilities in general:

There you have no chance to defend yourself or express your problem. The right of the employee does not exist. They only want you to work all the time. They don't like to listen to your problems. (Nurse, district hospital, FGD)

Another nurse in the public sector explained about health workers in church-run health services becoming ill:

If you are often sick you will definitely be dismissed, they will terminate the service. But in the government system they will accept you until you are cured. (Nurse, district hospital, FGD)

However, the perception of harsher disciplinary actions in the church-run health facilities was not shared by health workers at the church-run hospital. One staff member explained:

When an employee makes a mistake which deserves a reprimand, there is a special form for that, and when the mistake is made for a third time, then it is considered whether the employee should be dismissed. (Staff member in leadership position, church-run hospital, IDI)

Moreover, staff at the church-run hospital explained that the employee's trade union receives a copy of the formal warning.

### Work related benefits

The majority of the health workers interviewed reported that they would like to undergo further education as this would increase their knowledge and skills, and in most cases it would also mean a step towards promotion which in turn would lead to a higher salary and more prestige. One Clinical Officer in the church-run hospital explained:

Here there are opportunities for further training. This hospital tries its level best to allow people to advance. (CO, church-run hospital, IDI)

Staff in both the public health service and the church-run health service stated that the latter category of health workers has the best opportunities to receive further education funded by the employer. However, some health workers in the public sector argued that they have seen improvements in access to training in recent years. Moreover the emphasis on further training and availability of funds for staff development are likely to vary among church-run health facilities.

Another very important work-related benefit is attending seminars or workshops. The allowance paid to staff away from the workplace at seminars or workshops can constitute a substantial addition to their salary. The health workers interviewed in the church-run hospital argued that they have less access to attending seminars and workshops than employees in the public health services. A Nursing Officer at the church-run hospital with previous work experience from a public hospital argued:

I have worked here for some time and I have not yet gone to any seminar, but there [previous workplace], there were many seminars. But in the mission hospital you may work for five or seven years without attending any seminar. In the government, what they consider is your profession and whether the seminar is related to your sector. (Nursing Officer, church-run hospital, IDI)

The possibility of saving substantial amounts of the seminar allowance was mentioned by many of the informants as very important for their motivation.

The availability of housing of an acceptable quality was another factor emphasised by all the health workers interviewed. Health workers in the public health services argued that their staff quarters are generally of a poor quality. One Assistant Medical Officer argued:

For those of us who stay in the staff quarters, we feel that the quarters are normally in very poor conditions, it is actually a disgrace to stay in these quarters. (AMO, district hospital, FGD)

Health workers in the church-run hospital, in particular the female staff members, appreciated the safety of staying within the hospital compound. The quality of the housing in the compound of the church-run hospital was also emphasised. One Nursing Officer with substantial work experience at the church-run hospital explained:

We get good houses. From what we hear from the people who left this place and went to other working places, we have better housing here. (Staff member in leadership position (Nursing Officer), church-run hospital, IDI)

Staff members who stay outside the church-run hospital compound reported that they receive a housing allowance of 10% of their salary, a system which has no equivalent in the public sector.

### Work environment

In situations of severe resource constraints where health facilities are provided with far less equipment and drugs than required, health workers find that they are not able to provide the services expected. Where resources are available, health workers reported that they to a larger extent can assist the patients properly. Health workers in the church-run hospital frequently emphasised access to resources as being very important in providing good-quality health care, and maintained that this was an important aspect of their motivation to perform well. In the public health facilities health workers repeatedly pointed out that they experienced a lack of adequate infrastructure and equipment. They readily acknowledged that the quality of the services is better in the church-run hospital because of the health facility infrastructure and access to resources. One nurse with extensive work experience at the district hospital explained with reference to the church-run hospital:

There they have many machines which we don't have here. If we wish to examine special cases we have to send them where the special equipment is available. So if one works there it would help to gain higher experience, because there they can perform many operations. (Nurse, district hospital, IDI)

Health workers in the church-run hospital generally praised their hospital for its access to advanced medical equipment and other resources at the facilities. One staff member compared her current workplace with her experience of a hospital in another region and stated:

At a regional hospital in southern Tanzania, when patients came for medications, for every requirement, I had to write 'out of stock', 'out of stock'. Meaning that what I was doing was only talking, saying 'out of stock' and not supplying medications. At most what one could supply was paracetamol or aspirin. This made me despair and I got demotivated, because I would like to work to help and serve patients. (Staff member in leadership position, church-run hospital, IDI)

Another staff member explained her experiences from a regional hospital:

The things I saw there were disgusting and hard to explain. Any person who has worked in a mission hospital will not dare go into a government hospital. It is very difficult to work there because a patient is asked to bring along almost everything required for the medical services. Compared to this place, once the patient is brought to the hospital, the necessary services start up without being asked to bring anything. (Staff member in leadership position (Nursing Officer), church-run hospital, IDI)

In the church-run hospital a number of the health workers interviewed explained that the quality of the health services influenced their decisions on where they want to work. One staff member explained:

I like my job and I am really not after a big or high payment for what I do. I am interested in helping patients. Because however much they increase the salary, we have to provide proper services. If we are highly paid and the services we provide are not satisfactory, then surely we are not doing the right thing. (Staff member in leadership position, church-run hospital, IDI)

One staff member in the church-run hospital reflected on the reasons for not changing workplace:

I prefer working with a church-related health institution like this one. If I look at my own achievements and my experiences here, I am working much more smoothly than in the government where the shortages of working tools and equipment are rampant. (CO, church-run hospital, IDI)

Health workers in the church-run hospital praised the quality of services offered, and pointed out what they considered to be major differences from the public health sector where it was generally acknowledged that the resource constraints made it very difficult to provide good health care.

The interviewed health workers also pointed out the importance of values communicated by the hospital leadership for their motivation and attitudes towards the work. A staff member at the church-run hospital reflected on the meaningfulness of working in this type of health facility:

You can build yourself up spiritually. We start work with prayers and thus we find ourselves holistic. You find yourself close to the patients, and you can provide good service to them, both physically and spiritually. (CO, church-run hospital, IDI)

Many of the health workers interviewed from the church-run hospital emphasised the importance of the religious dimension of their work, and pointed to this as a factor influencing their decisions regarding workplace. This topic was not brought up among the health workers in the public health facilities.

### Personal/family concerns

Health workers need to make practical arrangements regarding where to establish their families and households. One staff member with previous work experience from the church-run hospital who had moved into the public health sector explained vividly the reason for coming back to the church-run hospital:

It is only the family which made me return [to the church-run hospital], as my husband was working here. I did return with a lot of bitterness. I had no other choice but to make that decision. I'll have to meet the challenges until his contract is over, then we can know what will be the next step. (Nursing Officer, church-run hospital, IDI)

Other female staff members similarly explained that their husbands' workplace was a determining factor in their choice of workplace. A related issue pointed out as being important to many health workers was living close to their extended family and the need to take care of elderly parents, siblings or other relatives.

### Concerns about pensions

The amount paid as a pension, particularly the lump sum, proved to be a major concern to all the health workers interviewed. Many viewed the lump sum paid upon retirement as an attractive financial bonus. One Nursing Officer at the district hospital explained:

In the government the pension benefits are actually good. A good amount of money is paid when one retires. (Nursing Officer, district hospital, IDI)

A medical attendant explained:

I think one has to stay in the government because it has many incentives, for example the pension benefits on retirement, which allow one retire comfortably. It is important to stay with the government and one should not attempt to go outside this system. (Medical attendant, district hospital, FGD)

A nurse explained about the pension scheme in a church-run hospital:

I preferred to come into the government services because the government pension is better. (Nurse, district hospital, FGD)

The health workers interviewed at the church-run hospital shared the view on the differences in the pension scheme. One staff member explained:

The thing which attracts people to the government, particularly the young people, is the pension scheme. In the government the retirement benefits are very high, compared to what is received in the church-related health institutions. This is the main factor making workers leave this place - hoping to get that relatively high pension payment. (Staff member in leadership position (Nursing Officer), church-run hospital, IDI)

All the interviewed health workers, regardless of current sector of employment and age emphasised the importance of the pension scheme and referred to the pension scheme as a determining factor for their preference for workplace. In practice, health workers who have accrued pension rights in one pension fund may fear to leave the employment because of the risk of loosing the pension rights. Younger staff members who can accrue full pension rights in another pension fund during the remaining part of their working life may be more likely to move to a health sector considered favourable.

The difference in the pension seemed to be a factor that strongly influenced health workers' evaluation of the workplace. Among all the health workers in both the public health sector and the church-run hospital there was a general perception that the pension schemes offered to the public sector employees were better than that offered in the church-run health facilities. Many of the health workers interviewed at the church-run hospital in fact explained that they would definitely prefer to work in the public health sector if they were newly educated, and the main reason given was the difference in the pension schemes.

The personal considerations behind decisions on where to work emerged as quite different for staff close to retirement age and younger staff expecting to work in the health sector for many years. Younger staff reported that their current employment was a stepping stone for further career development, and that they could easily move to other workplaces offering better conditions. Staff with long work experience focused more on the importance of maintaining the accrued pension rights and the burden of responsibility towards family, and found it difficult to change workplace. One Clinical Officer argued:

I have to consider the period which I have spent in the government service. By going into another health institution and starting afresh I will lose some of my benefits, such as retirement benefits. For me it is not advisable to leave my job now. (CO, dispensary, IDI)

In general, the closer to retirement age a person was, the more important continuing in the same sector was considered to be. Staff with extensive work experience at the church-run health facility also found that changing to a public health workplace was not possible. One staff member argued:

At my age, if you go into the government health service, you have to start afresh, starting from the beginning of the career as on first employment, while here I have already worked for 20 years. So I don't see any reason for leaving this place. (Staff member in leadership position (Nursing Officer), church-run hospital, IDI)

Although all health workers were concerned about the significant differences in the pension schemes, it was consistently argued that the closer to retirement age a person was the more limited were the opportunities to change workplace.

Our study confirms the pattern reported above of health workers leaving church-run health facilities for employment in the public sector. One Assistant Medical Officer with extensive work experience in the public sector explained:

Staff members previously moved from the government sector to the private and religious organisations. But recently the government improved its services, so the movement turned the other way around and people have started moving back to the government. (AMO, district hospital, IDI)

The reasons offered were largely related to the better pension scheme in the public sector.

## Discussion

The marked preference for public sector employment found in this study seems closely associated with the pension scheme. In a situation where church-run health facilities are vital in the health infrastructure in rural areas and where patients tend to prefer these services this represents a challenge to resource constrained health system. The preference for public sector employment is a result of careful weighing of considerations concerning working conditions. These considerations can be grouped in what we refer to as domains of concerns. One domain comprises health workers' rights in terms of salary, workload, working hours and pension schemes, as well as benefits such as access to allowances, housing and further training. The second domain comprises factors related to health workers' experience of work satisfaction in terms of access to resources and the possibility of providing good quality services in an environment emphasising religious and humanitarian dimensions. We argue that the first domain tend to pull health workers towards public sector employment whereas the second domain pull towards church-run health facilities. A third domain comprises a range of personal and family concerns which can counteract initial preferences and pull towards either sector.

Our data show that age and gender are important determinants for decisions on whether or not to change workplace. Long work experience seems to reduce the likelihood of health workers to change workplace. This mirrors the findings from a study in Uganda where Hagopian et al. found that "[o]lder respondents were more satisfied than younger ones ... Attachment to the facility and the community tended to be stronger with each older age group, and relationships with supervisors were better." [28]:w867. In our study we found that younger staff members are more likely to seek alternative employment. Wilson et al. argue that research to determine health workers' career intent is needed to understand factor that explain workplace preferences and issues of retention [29].

The health sector in Tanzania has been subject to significant reforms [30] and health workers' decisions regarding their workplace are also influenced by their experience of the changing policies and regulations [31]:1027. Leshabari et al. found that almost half of the doctors and nurses at the national hospital are not satisfied with their jobs and this is partly attributed to extensive reforms in the health sector [32]. Moreover, the difference in access to between public and church-run health facilities in reported a general phenomenon in Africa [33]. Gilson et al. argue that "[h]ealth policies and systems are complex social and political phenomena, constructed by human action rather than naturally occurring." [14]. Variation from one district to another and from one health facility to another is very likely and is a result of management style and the level of trust between employer and employees. Health workers in the public service express concerns about excessive disciplinary actions in the church-run health sector. However, the interviewed health workers in the church-run health facility did not find disciplinary action to be a concern in their employment. Negative perceptions of working conditions at other health facilities whether substantiated or not clearly influence decisions on workplace.

There are significant differences between Local Authorities Pension Fund (LAPF) and National Social Security Fund (NSSF) in terms of the pension paid. The pension schemes' websites [34,35] show that the government employees receive the overall best pension benefits and a considerably higher lump sum when retiring than employees in the church-run health sector. The lump sum offers opportunities that are perceived as very attractive due to the potential for investment, establishing a business or expanding an already-existing business. Concern about investments and thus securing the future is therefore the most likely explanatory factor behind health workers' strong focus on the pension schemes. In this context it appears rational that public sector health workers considered their current employment to be their best option. This is supported by the fact that many health workers in the church-run health facility stated that they would prefer to work in the public sector if they were newly educated and could decide on their workplace. Furthermore, the discourse on access to allowances is one prudent example of the concerns about the financial returns of the work, but as Ridde claims the allowance system may in effect undermine health systems [36].

The public health services in Tanzania are subject to serious challenges of resource constraints, and the Primary Health Services Development Programme 2007-2017 reports the shortcomings as "insufficient medical equipment, and shortage of medicines, supplies and laboratory reagents." [26]:5. Despite problems related to public sector working conditions [37], none of the health workers in the public health sector reported that they considered changing their employment to the church-run health sector. Although health workers generally expressed a clear preference, many remained in the same workplace because of already accrued pension rights and family concerns.

The domains of concerns identified in this study represent opportunities and constraints within which health workers navigate. This is important for health worker distribution and retention. Health workers' reasons for continuing to work at a workplace that offers a less favourable pension scheme may provide important information about factors influencing retention of health workers. Health workers in the church-run hospital were very concerned about the pension scheme, but downplayed its importance and emphasised the possibility of offering higher-quality health services as an important determinant for their decisions regarding workplace. Furthermore considerations reported by health workers, the practical implications of changing workplace in a rural setting may mean moving an entire household to a new location. The necessary investments are evaluated in relation to the expected benefits and inform health workers' decisions.

### How to improve retention of health workers in church-run health facilities?

The general trend in Tanzania is an increased movement of health workers from the generally better-equipped church-run health facilities to the public health services, mainly because of the differences in the pension schemes. It is important to address these issues through policies aimed at an equitable distribution of health workers. The importance of the financial aspects of working conditions has been reported in several studies. McCoy states that issues related to payment can "affect both retention within countries and distribution of health workers, whether between urban and rural areas or between public and private sector." [12]:675. Wyss argues that in order to counteract the negative consequences of health workers' movement between health facilities, "the use of monetary and nonmonetary incentives is of crucial importance for having the accurate skill mix at the appropriate place" [38]. In several African countries international NGOs attract local staff by offering salaries much higher than available to staff employed in the public service or other local employers. Regulation of this loss of staff has received increased attention in recent years [39,40] and on the local Tanzanian level, differences in employment conditions, including salary, allowance and pension, is an issue of major concern.

The differences and lack of coordination between the social security funds has recently received much attention, and an official report states that "[t]he social security sector lacks co-ordination at national level as each Fund reports to a different Ministry with differing operational rules and procedures. As a result, contribution rates, benefit structures, qualifying conditions as well as plans and priorities differ from one institution to another" [41]:9. The Trade Union Congress of Tanzania (TUCTA) has focused on the differences in the pension schemes and planned a strike in May 2010 [42]. The strike was, however, called off before it started. A Social Security Regulatory Authority Act was passed in 2008 and a Social Security Regulatory Authority (SSRA) has been established with a mandate to address the differences between the pension schemes [43]. Meanwhile the pension schemes available to only the 6% of the population formally employed [44]:12 will continue to affect decisions regarding workplace.

In a study in Tanzania, Kolstad found that continuing education is a powerful recruitment tool in attracting health workers to rural areas, but that salary and allowances are also very important. Housing, good infrastructure and the provision of equipment were other significant factors [45]. The pension scheme was not addressed in Kolstad's study but the factors identified as important for retention largely correspond with our findings on decisions regarding workplace. The factors identified in our study indicate that the retention of health workers and the distribution of health workers between the types of health facilities is a highly complex issue and much attention has been devoted to identifying measures to improve retention of health workers in rural areas. A WHO report lists six factors influencing decisions on workplace: 1) personal, 2) family and community, 3) financial aspects, 4) career related, 5) working and living conditions, and 6) bonding or mandatory service [3]:14. Huicho et al. likewise include similar issues as possible interventions to increase access to health workers in underserved areas [46]. The factors relevant for attracting health workers to rural areas by and large also apply to attracting and retaining staff at church-run health facilities. Lehmann et al. argue that "the development of appropriate strategies first requires an understanding of the factors which influence decisions to accept and/or stay in a remote post" [47]. Gilson and Erasmus point out the complexity of the retention of health workers and argue that "encouraging HRH retention requires a complex package of actions working through different entry points, rather than single policy actions" [10]:2. However, policies focusing on a single factor may also be effective provided that the improvement outweighs other factors. In a study in Malawi, Mangham and Hanson found that nurses were willing to trade between job attributes. Although the financial aspects of the working conditions were important, the study showed that nurses "were willing to forego pay increases for other improvements in their employment conditions" [48]. Couper et al. found in South Africa that a health worker's origin was a factor influencing decisions on whether to work in rural areas. In their study it was reported that health workers originating from rural areas are more likely to take up employment in rural areas and often in the home area for reasons of coming back to "roots, family, people, and village" [49].

The emphasis put on the pension scheme as a determining factor in our study indicates that health workers are responsive to interventions and efforts to influence the distribution and retention of health workers across health facilities. Retention not only concerns movement of health workers between types of health facilities but also whether the health sector is considered attractive for employment. Connell et al. argue that "[a] career in health is now seen as not having the prestige and salary it once had and nursing may be seen as a dirty, dangerous and difficult job whereas 'business' is the place of income generation, progress and action." [50]:1887. Policies aimed at correcting flaws in the distribution of health workers need to counteract negative sentiments, either through addressing the complexity of issues or through a focus on single factors facilitating acceptable trade-offs. Health workers are an important category of government employees, and offer their services to the population at large. Thus, their perceptions, experiences and movements in the labour market have to be observed with the greatest of interest.

## Conclusions

Health workers' experiences and perceptions of differences in working conditions have much influence on decisions on workplace. Many countries face challenges in attracting and retaining health workers in rural areas. This is also the case in Tanzania. In addition there is a trend of health workers leaving the church-run health facilities. Health workers' considerations for choice of workplace relate on the one hand to the rights of the health worker and on the other hand to professional commitment and responsibility for patient care. Health workers in the church-run services put much emphasis on the commitment to serving patients. Church-run health facilities are generally considered to offer better quality services than the public sector, hence attracting a disproportionate high number of patients. The differences in the pension scheme appear to be the single most important factor influencing decisions about workplace in the health services in Tanzania. The church-run health facilities are disadvantaged because the relevant pension scheme offers much lower pension benefits than that in the public health sector. The major factor counteracting this disadvantage was the access to material resources at church-run health facilities, which strengthened their capacity to provide higher-quality health care and a better working environment. A national regulation of the pension schemes is necessary intervention to ensure retention of qualified health workers at the rural church-run health facilities in Tanzania.

## Competing interests

The authors declare that they have no competing interests.

## Authors' contributions

NGS planned and designed the study under the supervision of AB. The data collection was carried out by NGS and DAM. The initial data analysis and development of the first drafts were carried out by NGS under the supervision of AB. KMM and DAM contributed substantially to subsequent versions and critically revised the manuscript. All authors read and approved the final manuscript.

## Pre-publication history

The pre-publication history for this paper can be accessed here:

http://www.biomedcentral.com/1472-6963/12/92/prepub
